# Anti-Smith Antibodies as a Predictive Factor for Developing Lupus Nephritis in Systemic Lupus Erythematosus Patients: A Systematic Review

**DOI:** 10.7759/cureus.66270

**Published:** 2024-08-06

**Authors:** Asra Saleem, Bushra Zeeshan, Gayanthi Dissanayake, Meaza Zergaw, Mohamed Elgendy, Alvin Billey

**Affiliations:** 1 Internal Medicine, California Institute of Behavioral Neurosciences & Psychology, Fairfield, USA; 2 Dermatology, California Institute of Behavioral Neurosciences & Psychology, Fairfield, USA; 3 Orthopaedics, California Institute of Behavioral Neurosciences & Psychology, Fairfield, USA; 4 Laboratory Medicine, California Institute of Behavioral Neurosciences & Psychology, Fairfield, USA

**Keywords:** kidney disease, nephritis, antibodies, lupus glomerular disease, sle, lupus nephritis, anti-smith antibodies

## Abstract

Lupus nephritis (LN) is the most frequent and lethal complication of systemic lupus erythematosus (SLE), often presenting with subtle or no initial symptoms. Therefore, it is crucial to identify SLE patients who are at risk of developing LN to ensure they receive timely intervention. Significant scientific efforts have been made to identify various genes and antibodies that could increase the risk of LN. Our objective is to review the role of anti-Smith antibodies in this disease and evaluate their potential as a predictive marker for LN. This review was done in accordance with the Preferred Reporting Items for Systematic Reviews and Meta-Analysis (PRISMA) guidelines. We searched for different study types from 2019 onwards as per our inclusion and exclusion criteria, to look for the significance of anti-Smith antibodies. The following databases were used: PubMed, PMC, Google Scholar, Science Direct, and Scopus. Twenty-two studies were checked for eligibility, of which 17 studies passed, based on the commonly used quality assessment tool for each of the corresponding studies. The study results indicated that anti-Smith antibodies are highly specific for SLE and are part of its classification criteria. In addition, we observed that positive titers correlate with disease activity. The presence of anti-Smith antibodies is influenced by ethnicity being most common among Black patients. However, the data regarding their effectiveness as a predictive marker for LN is not fully established. A more sensitive investigation and larger cohorts on diverse ethnic populations could provide a definitive answer regarding the role of anti-Smith antibodies in LN, highlighting the need for additional research.

## Introduction and background

Target tissues have deposits of immune complexes and many autoantibodies, which are hallmarks of the chronic multisystem autoimmune illness known as systemic lupus erythematosus (SLE), which has a wide spectrum of clinical manifestations [1,2]. There are numerous consequences associated with SLE, but the most prevalent one is lupus nephritis (LN), which affects the kidneys and raises mortality and morbidity rates [3,4].

Research has shown that effective treatment with immunosuppressants at the beginning of diagnosing LN, which occurs in about 50% of adult SLE patients, can improve the overall outcome [5-7]. Around 20% of these patients will progress to end-stage renal disease within five years of diagnosis, and 40% of those with LN will develop chronic kidney disease [7]. Although renal biopsy is a gold standard test for diagnosing and guiding the treatment, due to its invasive nature, its use remains limited [8-10]. This calls for detecting biomarkers capable of discerning lupus renal activity and its severity, foreseeing renal flares, and monitoring renal involvement in systemic SLE [1].

Over the last five years, significant advancements have been made in identifying and validating biomarkers for LN. Although antibody profile and its correlation with LN have been studied in the past, there is still no predictive model for LN. Traditional biomarkers like anti-dsDNA, proteinuria, hematuria, and creatinine have not been found to correlate with disease activity [9,10]. Previously, studies have reported that patients with positive anti-Smith antibodies are more likely to develop renal and central nervous system involvement [8]. Immune deposits with anti-Smith antibodies were found in the glomeruli of patients with SLE. 

We aim to investigate if anti-Smith antibodies can serve as a predictive factor in developing LN based on previous studies showing a potential to cause the disease pathology. Although data regarding the clinical significance of anti-Smith antibodies remains unknown, we further delve into its association with ethnicity and complement levels, which may also play a role in causing LN. The course and progression of LN are determined by an interplay of demographic factors such as race and ethnicity, histopathological features, and laboratory characteristics [7,11].

This review serves as a bridge to emphasize how an antibody can predict the onset of LN, allowing for the earlier initiation of treatment that can reduce morbidity and death in SLE patients.

## Review

Methods

This systematic review was performed based on the Preferred Reporting Items for Systematic Reviews and Meta-Analyses (PRISMA) 2020 guidelines [12].

Eligibility Criteria

Inclusion criteria: The systematic review includes patients with or without SLE and LN between the ages of 18-75. Data from the last five years from 2019 to 2024 were used. English, free full-text articles, and human studies were selected. Systematic reviews (SRs), with or without meta-analyses, cohort studies, case series, case-control, cross-sectional studies, case reports, editorials, and narrative reviews, were all included to reduce the risk of bias.

Exclusion criteria: Excluded are studies involving populations below 18 and above 75 years and animals. Studies exploring antibodies other than the anti-Smith antibodies in the title are not considered. In addition, studies published before 2019 or with unspecified publication dates and those in languages other than English without translations were excluded.

Search Strategy and Databases

The databases PubMed Central (PMC), PubMed, Science Direct, Google Scholar, and Scopus were explored in April 2024, for potential articles published from year 2019 onwards. Using the Boolean method, Medical Subject Heading (MeSH) terms were combined with keywords to identify all potentially relevant articles pertaining to the significance and predictable role of anti-Smith antibodies in populations above 18 years old. The data collection for this review was completed in May 2024.

The keywords used to conduct the search were "anti-Smith antibodies," "lupus nephritis," "lupus glomerular disease," "systemic lupus erythematosus," and "nephritis lupus." The details of the search strategy are listed in Table 1.

**Table 1:** Strategy of the bibliographic search in databases with their corresponding filters.

Databases	Keywords	Search strategy	Numbers of articles before filters	Filters	Search result
PubMed	Anti-Smith antibodies, lupus nephritis, lupus glomerular disease, nephritis lupus, systemic erythematosus lupus	Anti-Smith antibodies OR anti-ribonucleic protein antigen OR anti-ena antibody OR ena antibodies OR ribonuclear protein antibody OR rnp antibody OR (("anti-small nuclear ribonucleoproteins autoantibodies" [Majr]) AND "anti-small nuclear ribonucleoproteins autoantibodies" [Majr:NoExp]) AND "anti-small nuclear ribonucleoproteins autoantibodies" [Supplementary Concept:NoExp] AND lupus nephritis OR lupus glomerular disease OR nephritis-lupus OR ( "Lupus Nephritis/classification"[Mesh] OR "Lupus Nephritis/diagnosis"[Mesh] OR "Lupus Nephritis/epidemiology"[Mesh] OR "Lupus Nephritis/etiology"[Mesh] OR "Lupus Nephritis/immunology"[Mesh] OR "Lupus Nephritis/pathology"[Mesh] OR "Lupus Nephritis/physiopathology"[Mesh] ) AND systemic erythematous lupus OR lupus OR ( "Lupus Erythematosus, Systemic/complications"[Mesh] OR "Lupus Erythematosus, Systemic/diagnosis"[Mesh] OR "Lupus Erythematosus, Systemic/immunology"[Mesh] OR "Lupus Erythematosus, Systemic/pathology"[Mesh] OR "Lupus Erythematosus, Systemic/physiopathology"[Mesh] )	106893	Free full text, in the last 5 years, Humans, English, Adult: 18-75 years	1789
PMC	Anti-Smith antibodies, lupus nephritis, lupus glomerular disease, sle	Anti-Smith antibodies AND lupus nephritis AND SLE	872	Five years cutoff	540
Google Scholar	Anti-Smith antibodies, lupus nephritis, lupus glomerular disease, sle	“anti-Smith antibodies” AND “lupus nephritis” AND “SLE”	736	Five years cutoff	413
Science Direct	Anti-Smith antibodies, lupus nephritis, sle	“Anti-Smith antibodies” AND “lupus nephritis” OR “lupus glomerular disease” AND “SLE”	9372	Five years cutoff	383
Scopus	Anti-Smith antibodies, lupus nephritis, sle	Anti-Smith antibodies AND lupus nephritis AND sle	52	Five years cutoff, English	28

Selection of Studies

All references were exported to the EndNote reference manager, where they were grouped and alphabetized. The results yielded were reviewed by two independent reviewers for their applicability, and this was done by examining the title and abstract of the records. The duplicates were initially removed and then reviewed for titles and abstracts, excluding the irrelevant studies both manually and automatically. After retrieval, 22 free full-text articles were sought for quality appraisal and thus eligibility. Disagreements were resolved through discussion or consultation with a third reviewer. 

Quality Assessment and Risk of Bias

The full articles remaining were assessed for quality appraisal using the established criteria for individual studies. This was done by two independent reviewers. The qualities of studies were assessed using standardized tools such as the Newcastle-Ottawa Scale (NOS) [13]; case series by Joanna Briggs Institute (JBI) Critical Appraisal Checklist [14]; systematic reviews and meta-analyses, Assessment of Multiple Systematic Reviews 2 (AMSTAR 2) [15]; narrative reviews, Scale for the Assessment of Narrative Review Articles 2 (SANRA 2) [16]; and appraisal tool for Cross-Sectional Studies (AXIS) [17]. Each assessment tool had its criteria and different scoring. A point is given when a tool scores "YES" and "PARTIAL YES" or "1." Two points are given when "2" is indicated. For our review, a score of at least 70% for each assessment tool was accepted.

Results

**Figure 1:**
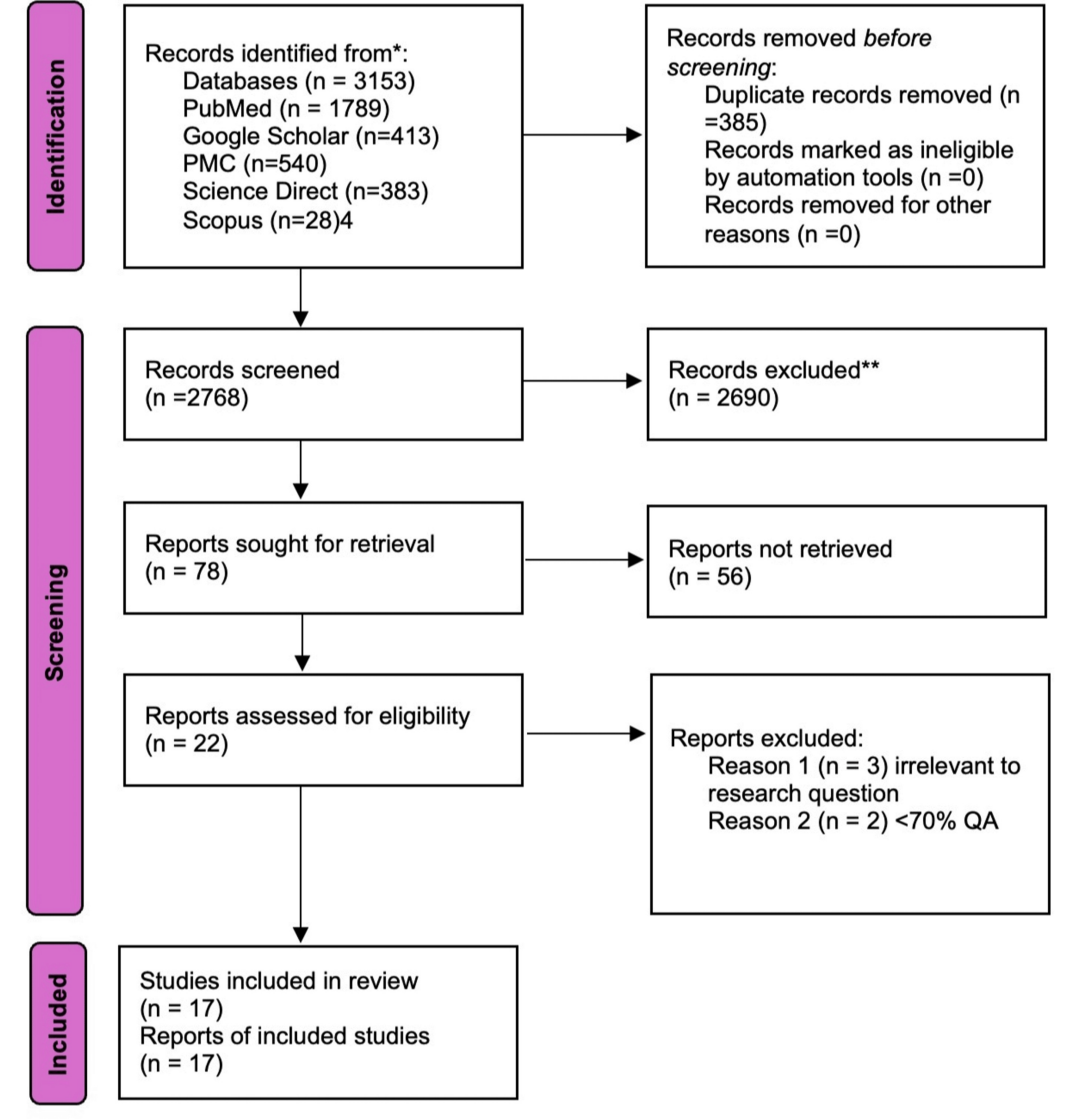
PRISMA flow diagram The Preferred Reporting Items for Systematic Reviews and Meta-Analyses (PRISMA) 2020 criteria served as the basis for this systematic review, which were used to screen and narrow down studies incorporated in this review [12].

The literature search identified 3,153 records. After removing duplicates, 2,768 unique records remained. Following the screening for title and abstract screening, 78 potentially relevant articles were sought for retrieval. Of these, 56 articles were excluded because of the inability to find free full text, leaving 22 studies suitable for the quality appraisal. Finally, the first author conducted a quality assessment of the retrieved reports, which was reviewed and approved by the second and third authors. Seventeen studies scored above 70% and were included in our study. These were two narrative reviews, nine observational studies including cohort and case-control, one randomized clinical trial, and six systematic reviews. Figure 1 outlines a flow diagram of the screening process and study selection.

Tables 2-6 demonstrate the features of different studies and their corresponding tools.

**Table 2:** Joanna Briggs Institute (JBI) for case series. Citation: [14]

First author, year	*Clear criteria for inclusion*	Condition measured in a standard, reliable way for all participants	Valid methods used for the identification of the condition for all participants	Consecutive inclusion of participants	Complete inclusion of participants	*Clear reporting of the demographics*	Clear reporting of clinical information of the participants	Outcomes clearly reported	Was statistical analysis appropriate?	Pass/fail
Elsayed, 2022 [8]	Yes	Yes	Yes	Yes	Yes	Yes	Yes	Yes	Yes	Pass
Sung soo ahan 2019 [18]	Yes	Yes	Yes	Yes	Yes	Yes	Yes	Yes	Yes	Pass
Yuan Fang, 2019 [19]	Yes	Yes	Yes	Yes	Yes	Yes	Yes	Yes	Yes	Pass

**Table 3:** Main characteristics of the systematic review using A Measurement Tool to Assess Systematic Reviews (AMSTAR) 2. NA: not applicable. Citation: [15]

Author, date	Item1	Item 2	Item 3	Item 4	Item 5	Item 6	Item 7	Item 8	Item 9	Item 10	Item 11	Item 12	Item 13	Item 14	Item 15	Item 16	Result
Guimarães, 2022 [6]	Yes	Yes	Yes	Yes	Yes	Yes	Yes	Yes	Yes	Yes	NA	NA	Yes	Yes	Yes	Yes	Included

**Table 4:** Appraisal tool for cross-sectional studies (AXIS). NA: not applicable. Citation: [17]

Author, date	Item 1	Item 2	Item 3	Item 4	Item 5	Item 6	Item 7	Item 8	Item 9	Item 10	Item 11	Item 12	Item 13	Item 14	Item 15	Item 16	Item 17	Item 18	Item 19	item 20	Results
Correa-Rodríguez Maria, 2021 [3]	Yes	Yes	Yes	Yes	Yes	Yes	No	Yes	Yes	Yes	Yes	Yes	NA	NA	Yes	Yes	Yes	Yes	No	Yes	Included
Zhang Yuxian, 2024 [20]	Yes	Yes	Yes	Yes	Yes	Yes	No	Yes	Yes	Yes	Yes	Yes	NA	NA	Yes	Yes	Yes	Yes	No	Yes	Included
Denvir Brendan, 2024 [21]	Yes	Yes	Yes	Yes	Yes	Yes	Yes	Yes	Yes	Yes	Yes	Yes	NA	NA	Yes	Yes	Yes	Yes	No	Yes	Included

**Table 5:** The main characteristics of narrative reviews using the Scale for the Assessment of Narrative Review Articles (SANRA 2) checklist based on six items. Five of the six studies scored above 70% that were included in our review. Citation: [16]

First author, year	Justification for the article’s importance for readership	Statement of concrete aims and formulation of questions	Description of the literature search	Referencing	Scientific reasoning	Appropriate presentation of data	Sum	Pass or fail
Maria Morell, 2021 [7]	2	1	0	2	2	2	9	Pass
David S. Pisetsky, 2020 [22]	2	1	1	2	2	1	9	Pass
Joyce J.B.C. van Beers,2022 [23]	2	1	1	2	2	1	9	Pass
Jan Damoiseaux 2021	2	1	1	2	1	1	8	Fail
Renaudineau Y, 2023 [24]	2	2	1	2	1	1	9	Pass
Alduraibi FK, 2024 [25]	2	2	1	2	2	1	10	Pass

**Table 6:** Findings of the Newcastle-Ottawa Scale (NOS) assessment tool for observational studies by review authors. Passing score: 7/9. Y: yes, N: no, NA: not applicable, NOS: Newcastle-Ottawa Scale Two out of eight studies failed, which were not included in our study. Citation: [13]

First author, year	Representativeness of exposed cohort	Selection of the non-exposed cohort	Ascertainment of exposure	Demonstration that outcome of interest was not present at the start of the study	Comparability of cohorts on the basis of the design or analysis	Assessment of outcome	Was the follow-up long enough for outcomes to occur?	Adequacy of follow-up of cohorts	Sum
Katelyn K. Bechler, BS, 2023 [5]	Yes	Yes	Yes	Yes	Yes	Yes	Yes	NA	8/9
Reppe Moe SE, 2019 [26]	Yes	Yes	Yes	Yes	Yes	Yes	Yes	Yes	8/9
Xuan Sun, 2024 [27]	Yes	Yes	Yes	Yes	Yes	Yes	Yes	NA	8/9
Choi, Hong Sang MD, 2019 [28]	Yes	Yes	Yes	Yes	Yes	Yes	Yes	NA	8/9
Terry Cheuk-Fung Yip, 2021 [29]	Yes	Yes	Yes	Yes	Yes	Yes	Yes	NA	7/9
Michelle Petri, MD, MPH, 2021	Yes	No	Yes	Yes	NA	Yes	Yes	Yes	6/9 fail
Dina F. Elessawi ^a^	Yes	No	Yes	Yes	Yes	NA	Yes	NA	6/9 fail
Jung-Min Shin, 2021 [30]	Yes	Yes	Yes	Yes	Yes	Yes	Yes	NA	8/9

Discussion

To comprehend the significance of our research question "Are anti-Smith antibodies a predictive factor for lupus nephritis?", it is essential to first understand the role anti-Smith antibodies play in SLE and the pathogenesis of LN. 

Pathogenesis of LN

LN can histologically be categorized into six classes, distinguished by the histological features and localization. Varied treatment strategies are employed depending on the stage of LN. The pathogenesis of lupus involves the development of autoantibodies that have long been recognized as key indicators of SLE [2,9]. These autoantibodies cause tissue damage through mechanisms like immune complex formation, binding to cell surfaces and causing cytotoxicity, reacting with autoantigens on activated or apoptotic cells, penetrating living cells, and combining with cross-reactive extracellular molecules [7]. Deposition of immune complexes and activation of the complement system causes intrarenal inflammation [7]. Besides triggering complement activation in the kidney, immune complexes can also stimulate the production of pro-inflammatory cytokines, particularly type I interferon [22]. Various stages of LN correlate with distinct serological markers. For instance, a predominant type 1 interferon signature is observed in kidneys afflicted with proliferative LN.

**Figure 2:**
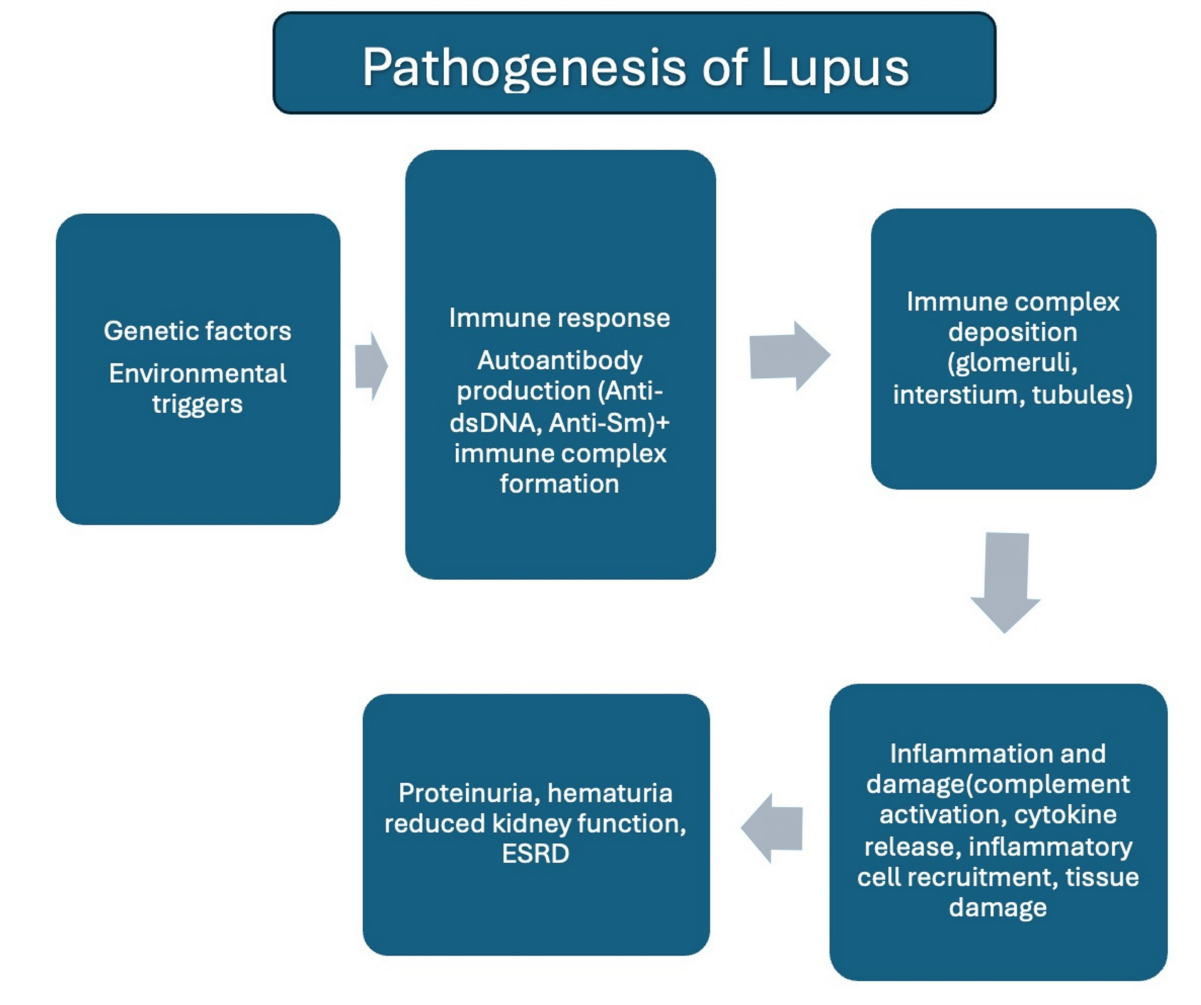
Pathogenesis of lupus nephritis. Anti-double stranded deoxyribonucleic acid (anti-dsDNA), anti-Smith (anti-Sm), end-stage renal disease (ESRD) Image Credits: Asra Saleem

While the term “lupus nephritis” implies a single type of disease, it encompasses a diverse range of kidney injuries affecting various tissue compartments of the kidney to different extents [6]. This results in clinical manifestations of varying severity and progression, making the discovery of reliable biomarkers particularly challenging. Identifying good biomarkers for LN is difficult due to the heterogeneity of the disease and its impact on different parts of the kidney with varying clinical outcomes. Figure 2 summarizes the pathogenesis of LN.

Antibodies and Their Associations

Although the serology in SLE can be complex, testing for ANA and anti-extractable nuclear antigens (anti-ENA) is crucial to ascertain the role of various antibodies in every patient [22]. These serological indicators are essential for the efficient treatment and monitoring of SLE patients. Of the many antibodies present in SLE, anti-Smith antibodies target proteins within the spliceosome complex. These antibodies are directed against seven proteins consisting of a small nuclear ribonucleoprotein (snRNP) particle core [18]. Studies show that the bone marrow of SLE patients contains more apoptotic cells, which may overexpose the nuclear antigens, such as the Smith antigen, which is a part of snRNPs involved in RNA processing [18,23]. They have a specificity of about 90% and are hence included in the SLE classification criteria [8,23]. Anti-Smith antibodies are particularly useful in diagnosing SLE, although their clinical relevance and correlation with illness symptoms remain unclear [23]. A positive test result of anti-Smith antibody strongly supports the diagnosis, but a negative test result cannot exclude it. There has been considerable interest in using ANA titers as predictors for clinal manifestations, disease progression, and primary site of activity in SLE to decrease its mortality and morbidity [7].

Anti-Smith Antibody Correlation With Disease Activity

**Figure 3:**
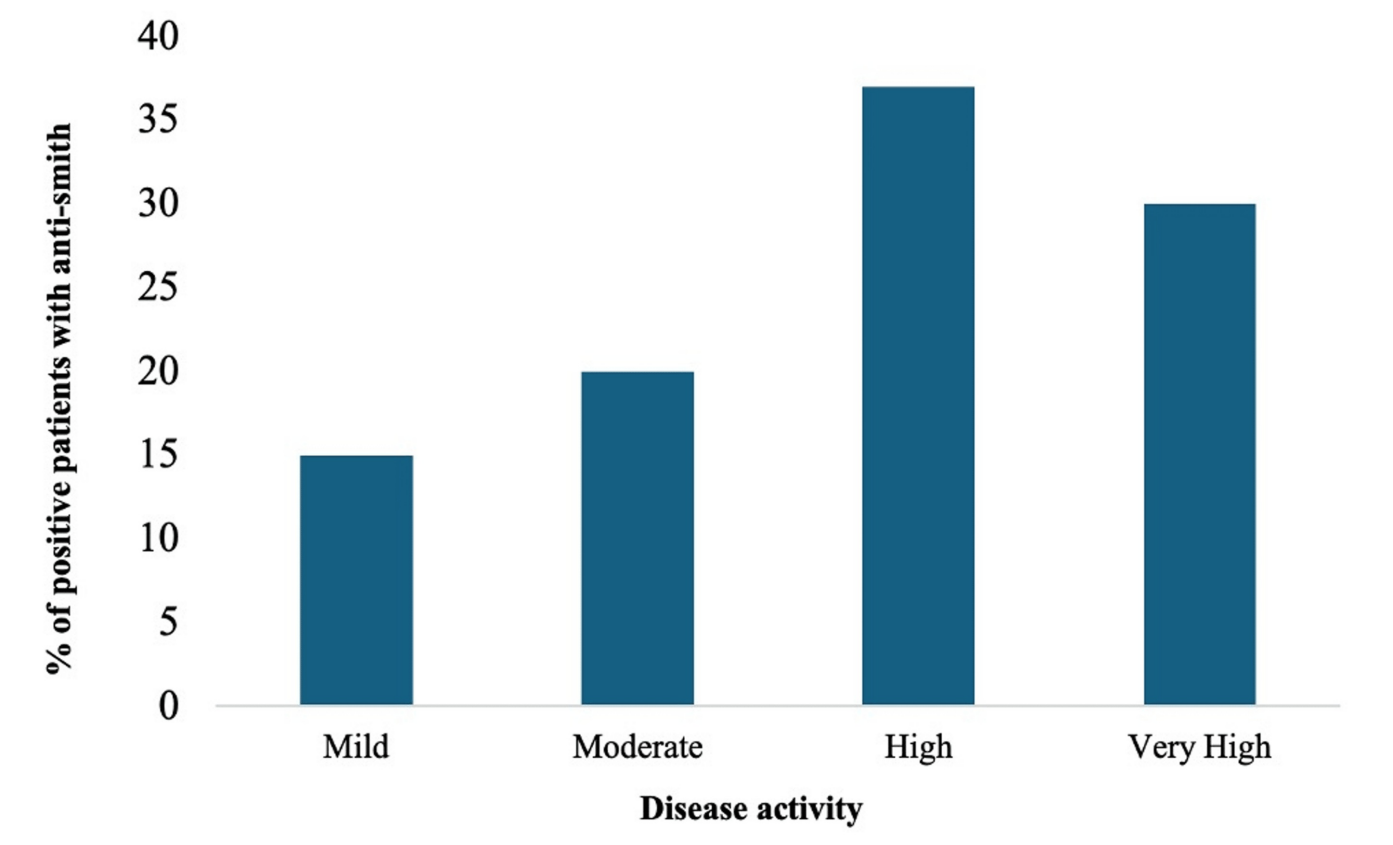
Distribution of anti-Smith antibodies in different grades of the Systemic Lupus Erythematosus Disease Activity Index 2000 (SLEDAI-2K) Image Credits: Asra Saleem

Studies that showed a positive correlation: The literature reveals that when the autoantibody positivity increased, the mean rank of SLE disease activity indexed 2000 (SLEDAI-2K) increased [18-19]. Sung Ahn in his study compared SLE patients with and without anti-Smith antibodies and found that those with anti-Smith antibodies were likely to have higher disease activity, represented by lymphopenia, hypocomplementemia, and higher SLEDAI-2 K. Variations in anti-Smith antibody titer may reflect changes in lupus disease activity, suggesting its potential as a serological marker for the disease activity in SLE patients [18]. In the same year, 2019, Fang Yuan, in his clinical research report, found that anti-Smith antibodies were a significant indicator of disease activity, as the titers of these antibodies were higher in patients with severe LN compared to those with mild LN, suggesting that higher levels correlate with severity of pathology [19]. A similar association of LN with anti-Smith was also found in a cohort study in Oslo [26]. This fact is further supported by Elsayed et al. (2022), who also found a positive correlation between anti-Smith and SLEDAI scores. In a recent cross-sectional study, patients with positive urinary anti-Smith antibody titers revealed 100% renal involvement, which was significantly higher compared to those with negative urinary anti-Smith antibody titers [20], which proves that in patients without initial renal involvement, a positive test for these antibodies may indicate future renal complications. Figure 3 shows a graphical representation of the positive correlation of anti-Smith antibodies with disease activity.

Studies that did not find a correlation between anti-Smith antibodies and LN: Correa-Rodríguez failed to detect significant associations between SLEDAI scores and anti-Smith antibodies, indicating that clinically detectable changes in disease activity do not correlate with these autoantibodies [3]. Moreover, a systematic review of the biomarkers for LN did not find anti-Smith antibodies to help detect LN [6]. In a recent study by Sun et al., the authors agreed that while specific antibodies may be detected in SLE, their clinical relevance in causing disease manifestations is dubious. Their research identified an anti-Smith antibody as one such antibody [27].

Analysis of Data and Discrepancies in the Results

The discrepancies in the results of different studies could be explained by the following: 1) Variability in study populations [28]: Different studies have examined various study populations of different racial and ethnic backgrounds, including differences in clinical features, disease activity index, and follow-up interval. 2) Small sample size [6]: Many studies have used a smaller sample size, which has resulted in an inability to detect true association by limiting its statistical power. 3) Heterogeneity of LN: As mentioned above, LN is a heterogeneous disease with multiple pathological forms that respond differently to anti-Smith antibodies. 4) Differences in the assay kit: It is noteworthy to state that the frequency of serological markers can vary depending on the assay kit used, underscoring the importance of further investigations to compare the results with those obtained using different assay kits

Due to the complex nature of SLE, including robust evidence of the influence of epigenetics, it is unsurprising that anti-Smith antibodies have yielded conflicting results in different studies [3].

Future studies must ascertain the antibodies' possible clinical importance because their longitudinal relationships and SLE clinical symptoms are mostly unclear.

Anti-Smith Antibodies Variation With Ethnicity

The occurrence of LN varies among different ethnicities and racial backgrounds, with some experiencing worse outcomes than others [21]. The presence of anti-Smith antibodies is also affected by ethnicity and sex [18,24]. These antibodies are more common among Black patients than White patients [24]. According to a recent study by Brendan Denver, non-Latino Black and Asian patients had higher proportions of anti-Smith antibodies than Latino and non-Latino White patients [21]. Furthermore, they discovered that in comparison to non-Latino White patients, a higher number of Latino patients tested positive for anti-Smith antibodies [21]. Evidence also indicates that individuals of African descent have a higher likelihood of producing anti-Smith and anti-RNP antibodies compared to those of European descent. The Hopkins lupus cohort study showed that African American patients with SLE had poor renal function and were less likely to improve their glomerular filtration rate (GFR) trajectory when compared to the Caucasian population [29]. Understanding of health disparities can be aided by knowledge of the distribution of antibody specificities among members of racial and ethnic minority groups.

Anti-Smith Antibodies and Complement System

Although the underlying mechanism of anti-Smith antibodies in causing disease activity is largely unknown, data suggest that it can promote complement activation in vivo [18]. Anti-Smith antibodies were found to be negatively correlated with complement levels [30]. Since complement activation is a crucial pathway in SLE pathogenesis, activating complement by anti-Smith antibodies may explain the relationship between these antibodies and disease activity in lupus [19,30]. Consumption of the complements leads to a diminished amount of serum component C3, which is linked to deterioration in renal histology and is a valuable indicator of the severity of LN. 

Anti-Smith and Genetics

**Figure 4:**
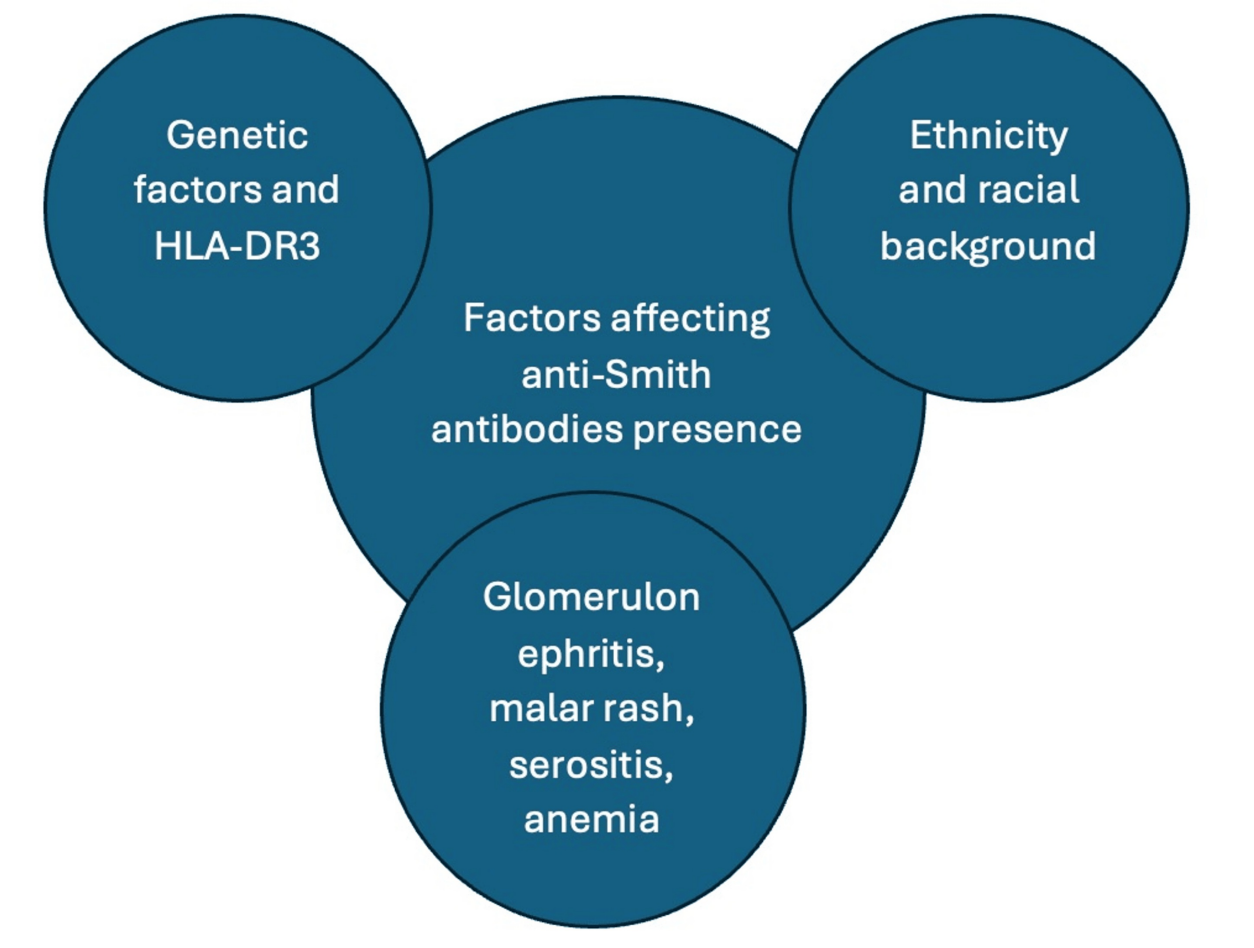
Factors affecting the presence of anti-Smith antibodies. Human leukocyte antigen–DR isotype 3 (HLA-DR3) Image Credits: Asra Saleem

The genetic components of LN have been of recent interest in the field of rheumatology, and we know from previous studies that genetics also contributes to the complex etiology of LN [30]. Specific genetic components have been linked to worse disease outcomes [25]. Human genetic research revealed a strong correlation between lupus and HLA-DR3 and HLA-DR15 across various ethnic groups [25,30]. HLA-DR3 is crucial in triggering an autoimmune response to anti-Smith antibodies and the development of LN, underscoring the importance of genetic factors in LN [24,30]. In a study conducted in 2021, it was postulated that the anti-Smith antibodies and low complement levels mediated an indirect relationship between weighted genetic risk score and LN [30]. A Korean cohort study found that individuals with the highest weighted genetic risk score (GRS) were independently associated with LN and the production of anti-Smith antibodies [25]. Although there have been advancements in this area, comprehensive research into the complete spectrum of genetic risk factors and their correlation with serological markers remains necessary for LN. Figure 4 highlights the factors affecting the presence of anti-Smith antibodies.

Predictive Value of Anti-Smith Antibodies

While anti-Smith antibodies increase the risk of developing LN in some patients, they are not universally predictive. Their presence alone as a single antibody cannot determine the renal outcome in SLE patients. 

Limitations

Our research has certain limitations that merit acknowledgment. With five databases, gray literature, and no study type limits, our overview covered a lot of ground. Nevertheless, the outcomes of an overview are vulnerable to errors brought on by data processing, even with the benefit of offering a broad and impartial perspective of the current information on a topic. First, our systematic review focused solely on articles published within the last five years, potentially excluding relevant information from earlier publications. Second, our analysis was restricted to articles that were freely accessible in the English language and available in full, possibly omitting valuable insights from sources we could not retrieve. Lastly, we excluded animal studies and pediatric populations, which could have provided invaluable perspectives on our research question.

## Conclusions

The results of our study show that anti-Smith antibodies are involved in the pathogenesis of LN in adult patients, and they can be used to indicate disease activity in LN but not as a predictive biomarker. Even while it is possible that these antibodies do not work well as BMs in LN, a more thorough investigation would yield conclusive evidence about their involvement in this illness. Future research endeavors could entail long-term monitoring of autoantibodies in larger cohorts of patients with SLE. In addition, including individuals from diverse ethnic backgrounds in these studies could offer valuable insights into the clinical significance of autoantibodies in SLE.
